# An improved fused feature residual network for 3D point cloud data

**DOI:** 10.3389/fncom.2023.1204445

**Published:** 2023-08-30

**Authors:** Abubakar Sulaiman Gezawa, Chibiao Liu, Heming Jia, Y. A. Nanehkaran, Mubarak S. Almutairi, Haruna Chiroma

**Affiliations:** ^1^College of Information Engineering, Fujian Key Lab of Agriculture IOT Application, Sanming University, Sanming, Fujian, China; ^2^Department of Software Engineering, School of Information Engineering, Yancheng Teachers University, Yancheng, Jiangsu, China; ^3^College of Computer Science and Engineering, University of Hafr Al-Batin, Hafar Al Batin, Saudi Arabia; ^4^College of Computer Science and Engineering Technology, Applied College, University of Hafr Al-Batin, Hafar Al Batin, Saudi Arabia

**Keywords:** point clouds, part segmentation, classification, shape features, 3D objects recognition

## Abstract

Point clouds have evolved into one of the most important data formats for 3D representation. It is becoming more popular as a result of the increasing affordability of acquisition equipment and growing usage in a variety of fields. Volumetric grid-based approaches are among the most successful models for processing point clouds because they fully preserve data granularity while additionally making use of point dependency. However, using lower order local estimate functions to close 3D objects, such as the piece-wise constant function, necessitated the use of a high-resolution grid in order to capture detailed features that demanded vast computational resources. This study proposes an improved fused feature network as well as a comprehensive framework for solving shape classification and segmentation tasks using a two-branch technique and feature learning. We begin by designing a feature encoding network with two distinct building blocks: layer skips within, batch normalization (BN), and rectified linear units (ReLU) in between. The purpose of using layer skips is to have fewer layers to propagate across, which will speed up the learning process and lower the effect of gradients vanishing. Furthermore, we develop a robust grid feature extraction module that consists of multiple convolution blocks accompanied by max-pooling to represent a hierarchical representation and extract features from an input grid. We overcome the grid size constraints by sampling a constant number of points in each grid using a simple K-points nearest neighbor (KNN) search, which aids in learning approximation functions in higher order. The proposed method outperforms or is comparable to state-of-the-art approaches in point cloud segmentation and classification tasks. In addition, a study of ablation is presented to show the effectiveness of the proposed method.

## 1. Introduction

Three-dimensional (3D) data are a great asset in the computer vision field since it contains detailed information on the whole geometry of detected objects and scenes. With the availability of massive 3D datasets and processing power, it is now possible to apply deep learning to learn specific tasks on 3D data such as segmentation with classification (Varga et al., 2020; Ergün and Sahillioglu, 2023; Qi et al., 2023), recognition, and correspondence (Long et al., 2021). There are several categories of 3D data representations including point cloud, voxel, mesh, multi views, octree, and many others. A comprehensive overview of point clouds and other 3D data representations may be found in the study by Bello et al. (2020) and Gezawa et al. (2020). Point cloud data processing employs a variety of approaches. Following dispatching a point cloud to a voxel grid that is quantized spatially in the grid space, volumetric models use a volumetric convolution to compute (Maturana and Scherer, 2015; Choy et al., 2016). Volumetric approaches correlate points with grid positions by using grids as data structuring technique and convolutional kernels in 3D to get data from nearby voxels. Although grid data structures are efficient, to maintain the granularity of the data position, a high voxel resolution is essential. The amount of processing and memory used grows in a cubical relationship with the voxel resolution since large point clouds are expensive to process. Furthermore, most point clouds contain ~90% empty voxels (Zhou and Tuzel, 2018), processing no data could use a lot of computing power. Point-based models are another type of point cloud data processing paradigm. Unlike volumetric models, point-based models offer effective computation but have poor data organization. For instance, PointNet (Charles et al., 2017) aggregates the data in the network's final stage using the point cloud without quantization, as a result the precise locations of the data are preserved. However, the cost of computation rises in lockstep with the point number. Subsequent studies (Qi et al., 2017; Wang et al., 2018; Yifan et al., 2018; Qiangeng et al., 2019; Wang Y. et al., 2019) aggregate information using a downsampling approach at each layer. Graph convolutional networks (GCN) have been used in the network layer to generate a local graph for each point cluster (Simonovsky and Komodakis, 2017; Kuangen et al., 2019; Wang L. et al., 2019; Li et al., 2023) that can be regarded as a variant of the PointNet++ design (Qi et al., 2017). This architecture, however, is costly in terms of data structuring [e.g., Random Point Sampling (RPS)]. As reported by Zhijian et al. (2019), data structuring costs account for up to 88% of the entire computational cost in three common point-based models (Li Y. et al., 2018; Yifan et al., 2018; Wang Y. et al., 2019). Furthermore, SO-Net (Li J. et al., 2018) employs the self-organizing map (SOM; Kohonen, 1998) to create a set of points used to model a point cloud's spatial pattern. Even though SO-Net considers a point cloud's regional correlation, SOM is trained independently. As a result, SOM's spatial modeling and a specific point cloud task are no longer coupled. DGCB-Net (Tian et al., 2020) uses cutting-edge convolutional layers built by weight-shared multiple-layer perceptrons (MLPs), to automatically extract local features from the point cloud graph structure. A feature aggregation is formed by concatenating the features received from all edge convolutional layers. Rather than stacking multiple layers deep, the DGCB-Net adopts a strategy to flatly extend point cloud feature aggregation.

**Figure 1:**
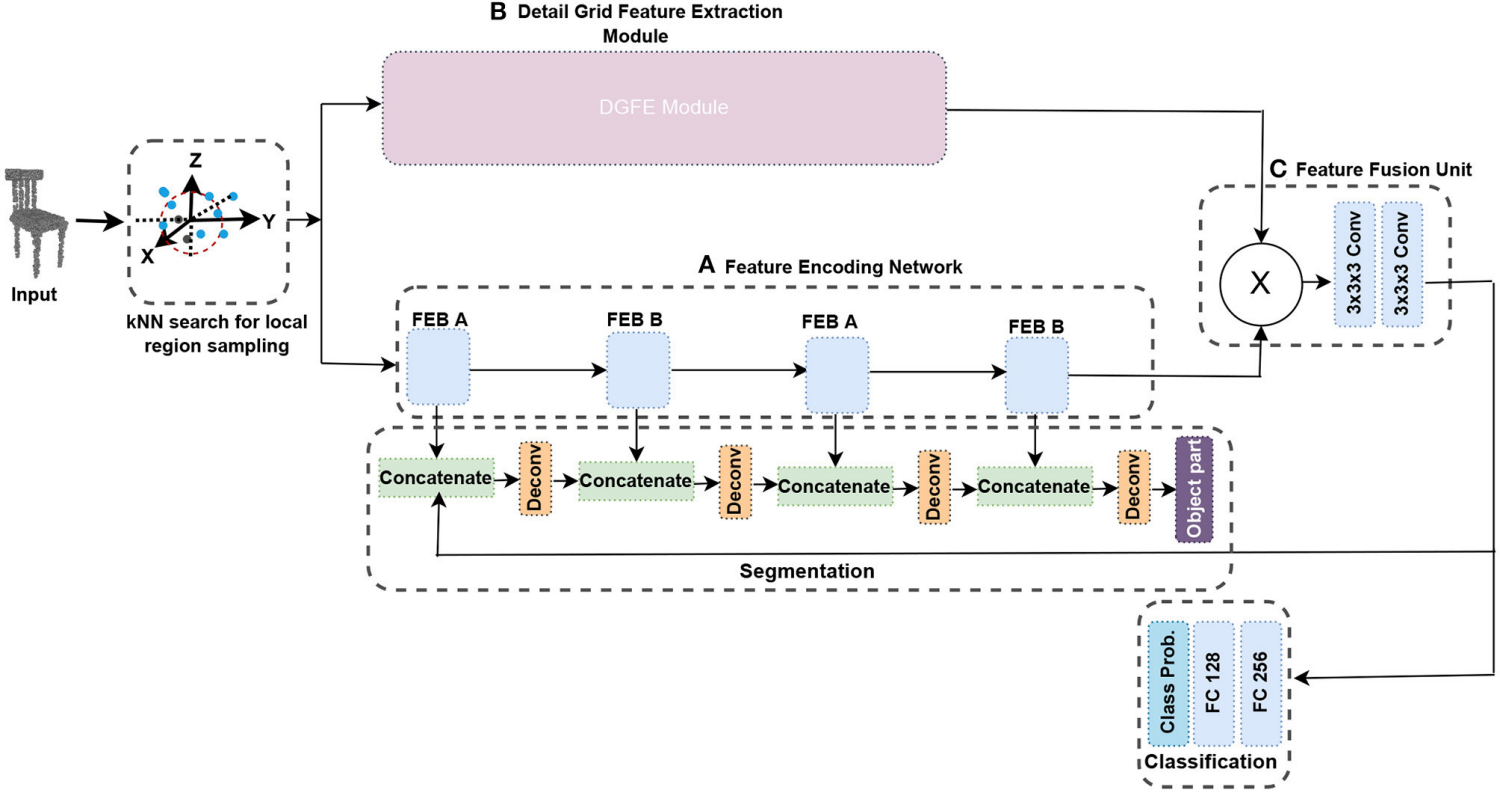
The complete architecture of the proposed method. The network is divided into three branches. The feature encoding network extract features from the input grid in **(A)**. The DGFE module exploits the detailed shape characteristics in **(B)**. The feature fusion unit which has two consecutive convolutional layers, fuses the features from the two branches to produce a feature with improved contextual representation by exploiting both local and global shape structures in **(C)**. See also Section 3.5.

In this study, we utilize deep learning to develop an approach that manage enormous 3D object datasets without compromising shape resolution. The majority of handcrafted 3D features are limited to low 3D resolutions. For example, Chiotellis et al. (2016) and Zhou and Tuzel (2018) require each 3D model in the datasets to be down-sampled to 20,000 faces with Meshlab before they can be fed into the system. Additionally, a method is provided that can handle structural variations in 3D objects without the need for data pre-processing. Many machine learning algorithms, such as the support vector machine (SVM), are effective when the datasets are small and well-curated, which implies that the data have been carefully pre-processed and requires human intervention. To address these challenges, this study offers an improved fused feature network, an end-to-end framework that solves shape classification and segmentation tasks using a two-branch technique with feature representation learning. To efficiently simplify the network, we start by developing a feature encoding network with two independent building blocks and layer skips with batch normalization and ReLU in between. Because there are few layers through which to propagate, using the layer skips speeds up learning and lessens the effect of gradients vanishing. Figure 1 presents the entire network structure of the approach. In addition, we create a detail grid feature extraction module, which comprises various convolution blocks accompanied by a max-pooling to represent a hierarchical representation of several feature representations and extracts features from the input grid. Max-pooling is used in each of the pooling layers, resulting in each spatial dimension having a smaller grid and helps to manage overfitting by gradually lowering the representation's spatial dimension, the parameters in the network, and the amount of processing. This module includes a regular-structured enclosing volumetric grid that helps capture details and features hierarchically. To extract features of high-resolution inputs, this module is utilized in conjunction with the feature encoding network. To pull through the limitation of the grid size, the local region in every grid sampled a constant number of points using a simple KNN search which aids in learning approximation functions in higher order to better characterize the details of the features.

Our major contributions are as follows:

- We design an effective module named detail grid feature extraction (DGFE) module. This module aids 3D convolutions to hierarchically capture global information and reduces the grid size in each spatial dimension as well as managing overfitting by gradually lowering the spatial dimension of the representation making it viable for high-resolution 3D objects.
- We design a feature encoding network that uses two different building blocks with layer skips containing batch normalization and ReLU in between, resulting in fewer layers in the early training phase which helps speed learning and reduces the effect of gradients vanishing since there are few layers through which to propagate.
- We built a network using the modules that have been proposed, which achieves a notable balance of accuracy and speed.

## 2. Related work

### 2.1. 3D learning using voxel-based methods

To build on the advance of CNN models on images (He et al., 2016a; Huang et al., 2017), Voxnet and its revisions (Maturana and Scherer, 2015; Wang and Posner, 2015; Wu et al., 2015; Brock et al., 2016) start by converting a point cloud to a grid occupancy and then used convolution in a volumetric form. To overcome the problem of rising memory usage due to cubical expansion, OctNet creates structures like a tree for non-empty voxels to avoid computing in space. While the volumetric approach is effective at structuring data, it suffers from poor computational effectiveness and data granularity loss. Transformers have lately been incorporated into the model designs of many 3D vision approaches in response to the success of transformer-based designs in the two-dimensional (2D) domain. The transformer has improved previous 3D learning techniques because of its ability to read remote input and provide task-specific inductive biases. The point-voxel transformer for single-stage 3D detection (PVT-SSD) proposed by Yang et al. (2023) uses input-dependent query initialization and voxel-based sparse convolutions for strong feature encoding. The PVT-SSD overcame the drawbacks of both point clouds and voxels by combining their advantages. To reduce farthest point sampling (FPS) runtime, they used sparse convolutions to transform points into a limited number of voxels rather than directly sampling them. They also sampled non-empty voxels. The voxel features were adaptively blended with the point features to make up for the difficulty of quantization.

### 2.2. 3D learning using point cloud-based methods

Charles et al. (2017); Qi et al. (2017) pioneered the use of point-based models which used pooling to aggregate the point features to achieve the permutation invariant. To better capture local characteristics, methods such as kernel correlation (Atzmon et al., 2018; Wu et al., 2019) and extended convolutions (Thomas et al., 2019) are proposed. To resolve the ambiguity, the local point order is predicted by PointCNN (Li Y. et al., 2018) while RSNet (Huang et al., 2018) sequentially consumes points from various directions. In methods based on points, the cost of computation grows linearly with the points input. The cost of structuring data, nevertheless, turned out to be a performance bottleneck for large inputs. Recently, a dynamic sparse voxel transformer (DSVT) was presented by Wang et al. (2023) in an effort to widen the uses of transformers so that they may serve as a solid foundation for outdoor 3D perception just as they do for 2D vision. A number of local regions are split up into smaller ones in each window using DSVT based on sparsity, and each window's attributes are then computed fully in parallel. Another recent point cloud classification framework named point content-based transformer (PointConT) was introduced by Liu et al. (2023), and it employs local self-attention in the space of features rather than the 3D space. One of the main advantages of PointConT is that it takes advantage of the locality of points in the feature space by clustering sampled points with similar features into the same class and computing self-attention within each class, allowing for an efficient trade-off between collecting long-range dependencies and computational complexity.

### 2.3. Strategies for point data structuring

The majority of point-based methods (Qi et al., 2017; Li Y. et al., 2018; Bello et al., 2021; Gezawa et al., 2021) employ FPS (Eldar et al., 1997) to sample uniformly distributed group centers. However, it does not account for the subsequent processing of the sampled points which may result in suboptimal performance. Random point sampling (RPS) has the advantage of having a minimal downtime. It is indeed, nevertheless, sensitive to variation in density. The KNN search we used for sampling the local region in each grid cell combines sampling and neighbor querying in a single step, making it faster than RPS.

SO-Net (Li J. et al., 2018), on the other hand, creates a self-organizing map. To split the spaces, KDNet (Klokov and Lempitsky, 2017) employs kd-tree. Gumble subset sampling is used instead of FPS by Yang et al. (2019). To create super points, Landrieu and Simonovsky (2018) employs a clustering algorithm. The majority of these approaches are either too slow or necessitate structure preprocessing. VoxelNet (Le and Duan, 2018; Zhou and Tuzel, 2018), for example, blends point-based and volumetric approaches by performing voxel convolution and employing the study by Charles et al. (2017) inside each voxel. Similar concepts are used by the fast model (Zhijian et al., 2019), whereas Lu et al. (2022) made use of ball query with graph convolution layers. However, the number of points is not steadily decreased over all layers. Our DGFE module, however, utilized max-pooling in each of the pooling layers, resulting in each spatial dimension having a smaller grid allowing it to be used for high-resolution 3D objects. Apart from those features, the local region in every grid sampled a constant number of points using a simple KNN search which aids in learning approximation functions in higher order to better characterize the detailed features.

## 3. The proposed method

In this section, the KNN search for local region sampling is first introduced. Following that, we propose the feature encoding network that serves as the basis of the enhanced fused feature network. The split-transform-merge paradigm, which is based on the residual learning framework, is one of the primary building block we employ to design our feature encoding network (Figure 1A). One of the primary benefits of employing the residual network is its simplicity in training networks with many layers without raising the training error percentage. It also aids in solving the vanishing gradient problem by applying identity mapping. To compensate for structural changes in 3D objects, our feature encoding network employs two different building blocks [feature encoding block (FEB) unit A and feature encoding block (FEB) unit B], with layer skips in between. We begin with 3x3x3 convolutions twice, followed by 1x1x1 convolutions with a stride in each convolution to accommodate both small and large datasets without possible overfitting and to lower the spatial dimension of the representation. Then, we introduce the detail grid feature extraction module and finally the feature fusion unit. The complete framework is presented Figure 1.

### 3.1. KNN search for local region sampling

Point clouds are typically represented as raw coordinates of points in 3D space. Here, we will go over how our model extracts features from 3D objects when given a point cloud of number of points (N) as input. When provided with an input of N × 3 set of point clouds, the object is then subdivided into equal-sized 3D voxels, such as 64 × 64 × 64, 16 × 16 × 16 or 8 × 8 × 8. Using KNN, K points will be sampled from each grid cell. To avoid extra computation, those with empty points will be padded with zeros. In contrast to standard KNN, in which the search area consists of all points, it just needs to search among non-empty voxels in our situation, making the query much faster. Unlike VoxNet (Maturana and Scherer, 2015) which represents the 3D structure using an occupancy grid, we build a grid from point clouds and designate the grid's key feature to the points that are inside each grid. Some grids, on the other hand, may contain a different point number. This implies that we need a grid that will share kernels in 3D convolution. Moreover, for addressing this constraint, we utilized a sampling strategy that ensures each grid has an equal point number. In particular, if there are beyond K points in the grid, we use the KNN sampling strategy to choose K points from the total points. K points are sampled with substitution when the points inside a grid are below K. Consequently, each grid will have the same number of points, allowing us to encode the grid feature so that each grid feature has the same feature size vector which enables us to extract hierarchical features of the object using 3D convolutional kernels.

### 3.2. Feature encoding network

We concentrate on developing a robust network for shape classification and segmentation that achieves a notable balance of accuracy and speed. The feature encoding network is one of the key blocks that we create by making use of the split-transform-merge paradigm, inspired by the residual learning framework design in the study by Szegedy et al. (2015), He et al. (2016a,b), and Elhassan et al. (2021) and leveraging its powerful representational ability. These networks are scalable structures that bundle building units with the same linked shape which are referred to as residual units or blocks. The original blocks in the study by He et al. (2016b) compute as follows:


(1) $$\begin{array}{l} O_{i}=h\left(I_{i}\right)+f\left(I_{i},Weights_{i}\right), \end{array}$$

(2) $$\begin{array}{l} I_{i+1}=f\left(O_{i}\right). \end{array}$$

In this case, *I*_*i*_ represents the i-th block's input feature. *Weights*_*i*_ = {*Weights*_*i*_, *k*∣1 ≤ *k* ≤ *K*} contains biases and weights connected to block i-th. K stands for total layers in a block. *f* signifies the block function, such as a pile of convolutional layers of two 3x3 in Equation 1. The operation following element-wise addition is represented by the function *f*, which is ReLU in Equation 1. The *h* function is designated as an identity mapping: *h*(*I*_*i*_) = *I*_*i*_. Similarly, if function *f* is identity mapping, *I*_*i*+1_≡*O*_*i*_. Putting Equation 2 into Equation 1 yields:


(3) $$\begin{array}{l} I_{i+1}=I_{i}+f\left(I_{i},Weights_{i}\right). \end{array}$$

**Figure 2:**
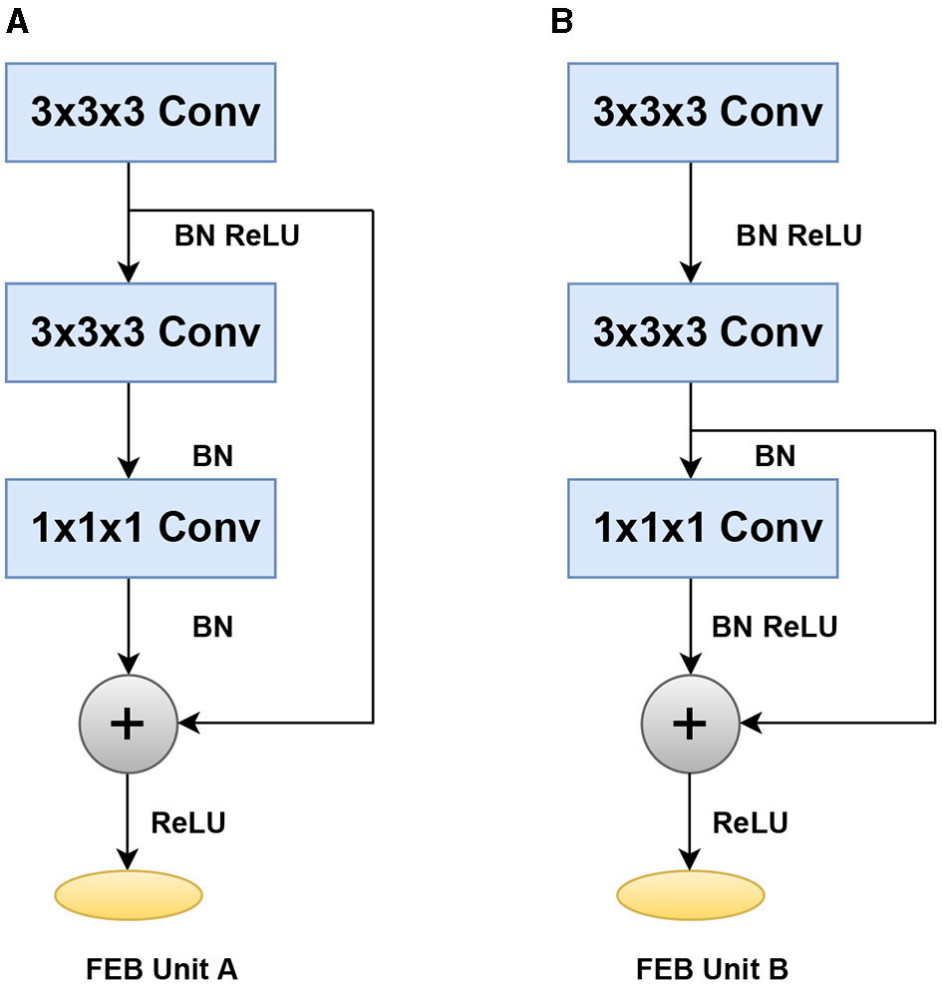
Building blocks of the feature encoding network with two different layer skips. **(A)** Feature encoding block (FEB unit A) **(B)** Feature encoding block (FEB unit B).

To efficiently accelerate training and reduce the number of parameters, the feature encoding network uses two separate construction blocks, such as Feature encoding block (FEB unit A) and feature encoding block (FEB unit B), with layer skips containing batch normalization (BN) and ReLU in between. The BN and ReLU are regarded as the weight layers' pre-activation, according to He et al. (2016b). We make some minor changes here by using the ReLu with BN and Conv before the addition of operation. We start with 3x3x3 convolutions twice, followed by 1x1x1 convolutions, and then we apply the BN and ReLu before the addition. We use a stride in each convolution to help manage overfitting by gradually reducing the spatial dimension of the representation. The feature encoding network's design is shown in Figure 2.

### 3.3. Detail grid feature extraction module

**Figure 3:**
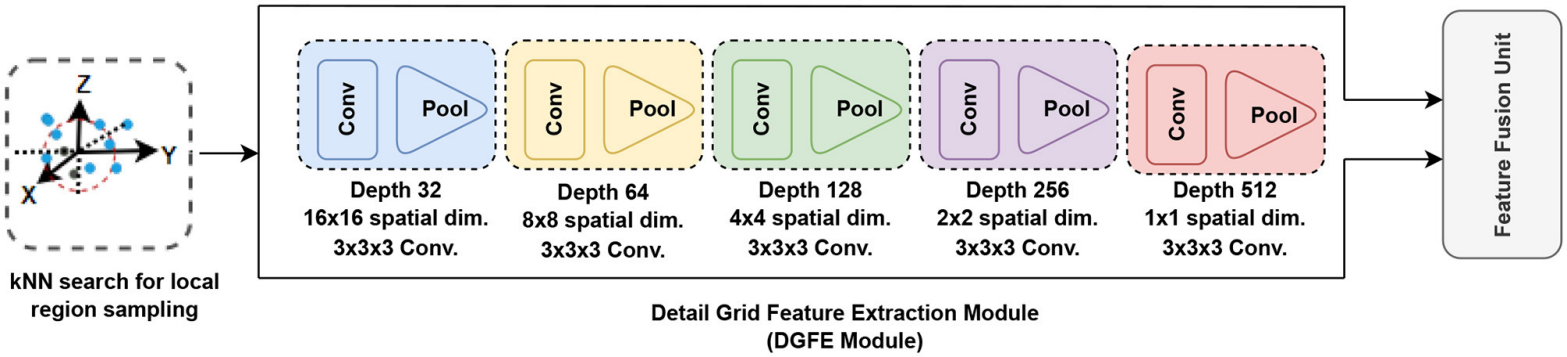
Detail grid feature extraction module (DGFE Module). This module extracts features from the input grid using many convolution blocks. Max-pooling is used in each of the pooling layers, resulting in each spatial dimension having a smaller grid and helps to manage overfitting by gradually lowering the representation's spatial dimension, the parameters in the network, and the amount of processing.

To represent numerous hierarchical feature representations, the detail grid feature extraction module employs several convolution blocks and max-pooling and extracts features from the input grid, as shown in Figure 3. Max-pooling is used in each of the pooling layers, resulting in each spatial dimension having a smaller grid and helps to manage overfitting by gradually lowering the representation's spatial dimension, the parameters in the network, and the amount of processing. BN (Ioffe and Szegedy, 2015) can be done to any set of network activations using:


(4) $$\begin{array}{l} y=g\left(Hu+p\right) \end{array}$$

where *H* and *p* are model parameters that have been learned, and *g*(.) denotes a non-linearity being ReLU or sigmoid. By normalizing *z* = *Hu*+*p*, the BN transform can be introduced right before the non-linearity. Since *z* is normalized, *y* = *g*(*Hu*+*p*) can be replaced with


(5) $$\begin{array}{l} y=g\left(BN\left(Hu\right)\right) \end{array}$$

where the BN (Ioffe and Szegedy, 2015) is used separately for each dimension of *z* = *Hu*, with a distinct set of learned parameters for each dimension. We utilized a 3 × 3 × 3 kernel with stride 1 convolution and a ReLU (Nair and Hinton, 2010) in each convolution layer. The initial block employs 32-filter convolutions, which are then doubled in subsequent blocks. This module offers a regular-structured embedding volumetric grid that supports 3D convolutions in hierarchically capturing global information. To extract features of high-resolution inputs, this module is utilized in conjunction with the feature encoding network. To keep local fine details in early encoder layers, at the same spatial resolution, we connect the encoder network's encoded features to equivalent features extracted from the detail grid feature extraction module.

### 3.4. Feature fusion unit

**Figure 4:**
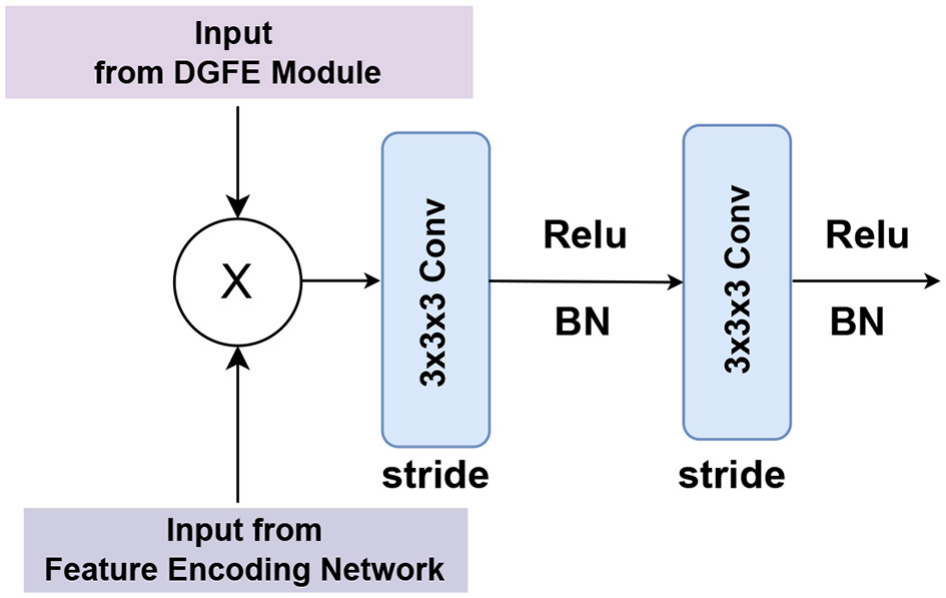
Illustration of the detailed design of the feature fusion unit, which consists of two consecutive 3x3x3 convolutions with BN and ReLU in between, as well as a stride in each convolution to help manage overfitting.

The feature fusion unit is made up of two consecutive convolutional layers. We used 3 × 3 × 3 convolutions twice, with BN and ReLU in between, and a stride in each convolution to help manage overfitting. The proposed DGFE module and the encoding network outputs are fused using a cross-product in the feature fusion unit, as shown in Figure 4, to produce a feature with improved contextual representation.

### 3.5. Network overview

We built a 3D convolutional network with fixed points inside each grid cell, which aids in the learning of local approximation functions in high-order that better capture local shape features. Figure 1 presents a diagram of the proposed architecture. The network is made up of two major modules. A feature encoding network that serves as the foundation for extracting features from the input grid, as shown in Figure 1A in Section 3.2, and detail grid feature extraction (DGFE) module which comprises various convolution blocks accompanied with an operation of max-pooling to help in representing several relational features and pull out features from the input (Section 3.3). We hierarchically combine these two modules to form the proposed improved fused feature network. The proposed DGFE module and the encoding network outputs are fused in the feature fusion unit containing two consecutive convolutional layers (Figure 1C) to produce a feature with improved contextual representation by utilizing both local and global shape structures.

The point cloud is first normalized within the unit box. In each grid, the coordinates of the points are piled as features. accordingly, given the appropriate x, y, and z coordinates, a K-point grid has features 3K. In theory, by dividing the sum of points (P) by its grid cells, K can be approximated. To acquire classification scores, the resulting fused feature can be categorized using two fully connected layers. Finally, one additional fully connected layer is added, along with a softmax, which aids in regressing the likelihood in every group. The whole layer's nodes correspond to the set of categories of objects inside the dataset. To generate the segmentation, the segmentation network decodes the retrieved features. To create the output, this network upsamples and combines the features. For every cell inside the grid, this network produces K+1 labels, as for K points in that cell equivalent to K labels and one more label level cell. Obtaining ground truth labels of object components, we chose its greater label among the labels of points within every cell. Unoccupied Cells are tagged "no label." Before actually acquiring the object part, we perform a deconvolution operation by concatenating the feature obtained from the feature fusion unit, with the feature retrieved out of each block of the feature encoding network.

## 4. Experiments

In this section, a number of datasets including ModelNet10 and ModelNet40 (Wu et al., 2015) for object classification and part segmentation on ShapeNetPart (Yi et al., 2016) were used to assess the performance of the proposed network. We discuss the dataset's specifics and the evaluation metrics in Section 4.1. The implementation protocol discussion presented in Section 4.2. In Sections 4.3, 4.4, and 4.5, we discuss some experimental results from applying the proposed network to classify shapes on ModelNet, measure precision-recall on ModelNet10, and segment parts on ShapeNetPart. In Section 4.6, we demonstrate the advantages of the proposed method by conducting a good set of ablation experimental tests to evaluate various setup adjustments.

### 4.1. Datasets and evaluation metrics

**ModelNet dataset:** This is indeed a notable dataset. It comprises two datasets with CAD models in 10 and 40 categories, respectively. ModelNet10 is made up of 4,899 object instances including 2,468 training samples and 909 testing samples. ModelNet40 is made up of 12,311 object instances, 9,843 of which are in the training set and 3,991 samples in the testing set. For object classification on the ModelNet dataset, we employed accuracy as the assessment metric.

**ShapeNetPart dataset:** There are 16,881 shapes in this dataset, divided into 16 categories and annotated with a combined amount of 50 components. A considerable share of shape categories is partitioned into 2–5 segments. We, then, used mean intersection over union (mIoU) for evaluation. For every part shape within the object category, we calculate the union of prediction and ground truth. The mIoU was computed using Equation 6 as follows:


(6) $$\begin{array}{l} mIoU=\frac{X}{X+G-P} \end{array}$$

where *G*, *P*, and *X* denote the number of ground truth points, predicted positive points, and true positive points, respectively. The mIoU is obtained by taking the average of each class's IoU.

### 4.2. Implementation protocol

In Python, the proposed method was implemented using the Tensorflow deep learning library. Each experiment is conducted on an Nvidia Geforce Titan GTX GPU, CUDA 10.1, and CuDNN 7.1 with RAM of 12 GB. For the classification task, we test with various parameters setup including different grid sizes and K values. Each point's location is jittered with a standard deviation of 0.02. The batch size is 32, and batch normalization is used for all layers. For both the segmentation and classification tasks, we used the cross-entropy loss to improve the discrimination of the class features. We utilized an initial learning rate of 10^−4^ and employ Adam optimizer (Kingma and Ba, 2015).

**Loss function:** Over the years, a wide range of loss functions have been proposed to perform 3D shape analysis tasks. For example, the cross-entropy loss was already been utilized successfully in many shape analysis tasks. Although the network can be trained using cross-entropy loss alone, we employ a combination of Shape loss (Wei et al., 2020) and modified cross-entropy loss (Huang et al., 2019) to make the class features more discriminatory. The Shape Loss is given as follows:


(7) $$\begin{array}{l} L_{shape}=L_{s}\left(C\left(S\right),M\right) \end{array}$$

where *M* is the shapes's class label, *L*_*s*_ is a cross-entropy loss based on shape feature *S*, and *C* is a classifier.

Moreover, the cross-entropy loss is given as follows:


(8) $$\begin{array}{l} L_{cross-entropy}=\frac{1}{n}\underset{y}{\sum }\left(zlogQ+\left(1-z\right)log\left(1-Q\right)\right) \end{array}$$

For each sample, *Q*∈[0, 1] is the likelihood of the network output and *z* represents the class ground truth. To minimize the weight of easily categorized samples, the cross-entropy function can be reshaped by inserting a hyperparameter that aids in weight balancing.


(9) $$\begin{array}{l} L_{cross-entropy}=\frac{1}{n}\underset{y}{\sum }\left[z{\left(1-Q\right)}^{\gamma }logQ+\left(1-Q\right)Q^{\gamma }log\left(1-Q\right)\right] \end{array}$$

Once a sample is successfully identified, $Q\overset{}{\to }1$, the factor $\left(1-Q\right)\overset{}{\to }0$; Alternatively, when *Q* is small, the factor (1−*Q*) approaches 1. Our total loss is the combination of this two losses as follows:


(10) $$\begin{array}{l} L_{total}=L_{shape}+L_{cross-entropy} \end{array}$$

### 4.3. Classification on ModelNet

We use the PointNet (Charles et al., 2017) convention to prepare the data. Input points are set to 1,024 by default. Furthermore, we improve performance by incorporating more points and surface normal. To analyze various models to varying degrees of speed and accuracy, the network is trained with varying settings to balance speed and performance (Section 4.6). The variants are in different grid sizes and K values.

#### 4.3.1. Classification on ModelNet10

**Comparison:** The proposed improved fused feature residual network approach was compared with a number of state-of-the-art methods, as shown in Table 1. The proposed method outperforms the majority of previous voxel-based techniques in terms of "overall accuracy" including VoxNet (Maturana and Scherer, 2015), 3DShapeNets (Wu et al., 2015), 3DGAN (Wu et al., 2016), VSL (Liu et al., 2018), and BV-CNN's (Ma et al., 2017). Although VRN (Brock et al., 2016), which combines many networks, outperforms our method in ModelNet classification, their network structure is quite complex, with each network being trained separately and taking many days to complete, making them unsuitable for large datasets. When compared with point cloud-based methods, the proposed method outperforms many of them, including Dominguez et al. (2018), OctNet (Riegler et al., 2017), ECC (Simonovsky and Komodakis, 2017), DGCB-Net (Tian et al., 2020), and VACWGAN-GP (Ergün and Sahillioglu, 2023). The DGFE module helps 3D convolutions hierarchically acquire global information, allowing the network to capture the contextual neighborhood of points. Despite using viewpoints in a predefined sequence, as opposed to any random views by DeepPano (Shi et al., 2015), Gan classifier (Varga et al., 2020), GPSP-DWRN (Long et al., 2021), OrthographicNet (Kasaei, 2019), PANORAMA-NN (Sfikas et al., 2017), and SeqViews2SeqLabels (Han et al., 2019) both of which are multi-view techniques, the method outperforms these approaches, making it suitable for high resolution input. The proposed method also outperforms PolyNet (Yavartanoo et al., 2021), a mesh-based 3D representation network that combined the features in a much smaller dimension using PolyShape's multi-resolution structure.

**Table 1:** Object classification accuracy (%) on ModelNet10.

**Method**	**Input**	**Acc (%)**
VoxNet (Maturana and Scherer, 2015)	Volume	92.0
3DShapeNet (Wu et al., 2015)	Volume	83.5
3DGAN (Wu et al., 2016)	Volume	91.0
VSL (Liu et al., 2018)	Volume	91.0
BV-CNNs (Ma et al., 2017)	Volume	92.3
VRN (Brock et al., 2016)	Volume	97.1
PolyNet (Yavartanoo et al., 2021)	Mesh	94.9
DeepPano (Shi et al., 2015)	Image	85.4
OrthographicNet (Kasaei, 2019)	Image	88.5
PANORAMA-NN (Sfikas et al., 2017)	Image	91.1
SeqViews2SeqLabels (Han et al., 2019)	Image	94.8
Geometry-image (Sinha et al., 2016)	Image	88.4
Gan Classifier (Varga et al., 2020)	Image	89.2
GPSP-DWRN (Long et al., 2021)	Image	92.4
G3DNet (Dominguez et al., 2018)	Point	93.1
OctNet (Riegler et al., 2017)	Point	90.4
ECC (Simonovsky and Komodakis, 2017)	Point	90.0
DGCB-Net (Tian et al., 2020)	Point	94.6
VACWGAN-GP (Ergün and Sahillioglu, 2023)	Point	91.7
(Ours)	Point	**95.6**

The bold values used to differentiate our results from the rest of the other methods.

#### 4.3.2. Classification on ModelNet40

**Comparison:** We further tested the effectiveness and applicability of the proposed approach using the ModelNet40 dataset. Table 2 compares the classification accuracy of the proposed method to that of alternative scalable 3D representations techniques on the ModelNet40 datasets. As observed, the proposed method performs better than VoxNet (Maturana and Scherer, 2015), 3DGAN (Wu et al., 2016), 3DShapeNets (Wu et al., 2015), NormalNet, VACWGAN-GP (Wang et al., 2019a; Ergün and Sahillioglu, 2023), DPRNet (Arshad et al., 2019), Pointwise (Hua et al., 2018), BV-CNN's (Ma et al., 2017), NPCEM (Song et al., 2020), ECC (Simonovsky and Komodakis, 2017), PointNet (Charles et al., 2017), Geometry image (Sinha et al., 2016), VSL (Liu et al., 2018), GIFT (Bai et al., 2016), FPNN (Li et al., 2016), DGCB-Net (Tian et al., 2020), and DeepNN (Gao et al., 2022) that utilized mesh 3D data. The recent RECON (Qi et al., 2023) and PointConT (Liu et al., 2023) slightly outperformed our technique, which could be attributed to their usage of transformers and pre-train models. The improved fused feature residual network offers a significant advantage over the bulk of voxel and point cloud-based approaches, as shown in Table 2. The proposed method performs below VRN (Brock et al., 2016), which makes usage of 24 rotating replicas for training and voting when compared with non-voxel-based approaches. Additionally, the proposed method outperformed PolyNet (Yavartanoo et al., 2021), a mesh-based 3D representation network that integrated the features in a much fewer dimension using PolyShape's multi-resolution structure. It is also worth noting that the improved fused feature residual network proposed already has a high level of accuracy, with a score of above 90%. This may be attributed to the fact that our feature encoding network together with the DGFE module, directly extracts features from the input grid and represents an organized structure of numerous feature representations.

**Table 2:** Object classification accuracy (%) on ModelNet40.

**Method**	**Input**	**Acc (%)**
VoxNet (Maturana and Scherer, 2015)	Volume	83.0
3DShapeNet (Wu et al., 2015)	Volume	77.0
3DGAN (Wu et al., 2016)	Volume	83.3
VSL (Liu et al., 2018)	Volume	84.5
BV-CNNs (Ma et al., 2017)	Volume	85.4
VRN (Brock et al., 2016)	Volume	95.5
NormalNet (Wang et al., 2019a)	Volume	88.6
DeepNN (Gao et al., 2022)	Mesh	91.0
PolyNet (Yavartanoo et al., 2021)	Mesh	82.8
GIFT (Bai et al., 2016)	Image	83.1
DeepPano (Shi et al., 2015)	Image	77.6
OrthographicNet (Kasaei, 2019)	Image	88.5
SeqViews2SeqLabels (Han et al., 2019)	Image	93.0
Geometry-image (Sinha et al., 2016)	Image	83.9
PointNet (Charles et al., 2017)	Point	89.2
PointConT (Liu et al., 2023)	Points	93.5
RECON (Qi et al., 2023)	Point	93.9
Pointwise (Hua et al., 2018)	Point	86.1
NPCEM (Song et al., 2020)	Point	89.4
ECC (Simonovsky and Komodakis, 2017)	Point	83.2
DGCB-Net (Tian et al., 2020)	Point	92.9
3DCTN (Lu et al., 2022)	Point	91.2
VACWGAN-GP (Ergün and Sahillioglu, 2023)	Point	81.3
(Ours)	Point	**93.1**

The bold values used to differentiate our results from the rest of the other methods.

#### 4.3.3. ModelNet40 per-class classification accuracy comparison

**Figure 5:**
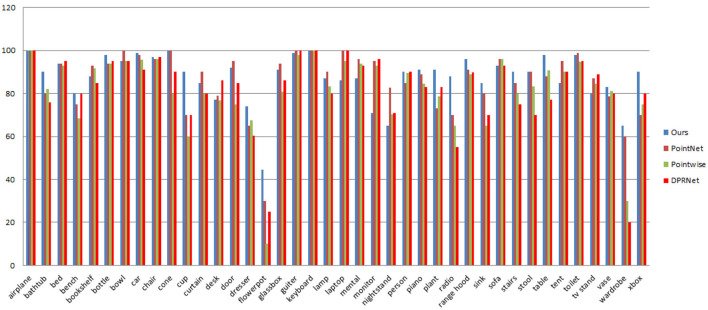
ModelNet40 per-class classification accuracy comparisons between PointNet, Pointwise, DPRNet, and (proposed).

Table 3 and Figure 5 compared the per-class accuracies of the proposed method to PointNet (Charles et al., 2017), Pointwise (Hua et al., 2018), and DPRNet (Arshad et al., 2019) on ModelNet40 dataset. As shown in Table 3 and Figure 5, using residual learning and extracting detail features improves per class classification accuracy. The proposed method outperforms PointNet, Pointwise, and DPRNet in key classes such as bathhub, car, bottle dresser, flowerpot, cup, and radio. In terms of average class performance, the method outperformed PointNet (1.2%), Pointwise (6%), and DPRNet (5.5%). Table 3 illustrates it.

**Table 3:** ModelNet40 per-class classification comparison between PointNet, Pointwise, DPRNet, and (ours).

**Methods**	**Ours**	**PointNet**	**Pointwise**	**DPRNet**
**Avg. class**	**87.4**	**86.2**	**81.4**	**81.9**
Airplane	100	100	100	100
Bathtub	90.0	80.0	82.0	76.0
Bed	94.0	94.0	93.0	95.0
Bench	80.0	75.0	68.4	80.0
Bookshelf	88.0	93.0	91.8	85.0
Bottle	98.0	94.0	93.9	95.0
Bowl	95.0	100	95.0	95.0
Car	99.0	97.9	95.6	91.0
Chair	97.0	96.0	96.0	97.0
Cone	100	100	80.0	90.0
Cup	90.0	70.0	60.0	70.0
Curtain	85.0	90.0	80.0	80.0
Desk	77.0	79.0	76.7	86.0
Door	92.0	95.0	75.0	85.0
Dresser	74.0	65.1	67.4	60.5
Flowerpot	44.6	30.0	10.0	25.0
Glassbox	91.0	94.0	80.8	86.0
Guiter	99.0	100	98.0	100
Keyboard	100	100	100	100
Lamp	87.0	90.0	83.3	80.0
Laptop	86.0	100	95.0	100
Mental	87.0	96.0	93.9	93.0
Monitor	71.0	95.0	92.9	96.0
Nightstand	65.0	82.6	70.2	70.9
Person	90.0	85.0	89.5	90.0
Piano	91.0	88.8	84.5	83.0
Plant	91.0	73.0	78.8	83.0
Radio	88.0	70.0	65.0	55.0
Range hood	96.0	91.0	88.9	89.9
Sink	85.0	80.0	65.0	70.0
Sofa	93.0	96.0	96.0	93.0
Stairs	90.0	85.0	80.0	75.0
Stool	90.0	90.0	83.3	70.0
Table	98.0	88.0	90.9	77.0
Tent	85.0	95.0	90.0	90.0
Toilet	98.0	99.0	94.9	95.0
TV stand	80.0	87.0	84.5	89.0
Vase	83.0	78.8	81.3	80.0
Wardrobe	65.0	60.0	30.0	20.0
Xbox	90.0	70.0	75.0	80.0

The bold values used to differentiate our results from the rest of the other methods.

### 4.4. Precision-recall on ModelNet10

**Figure 6:**
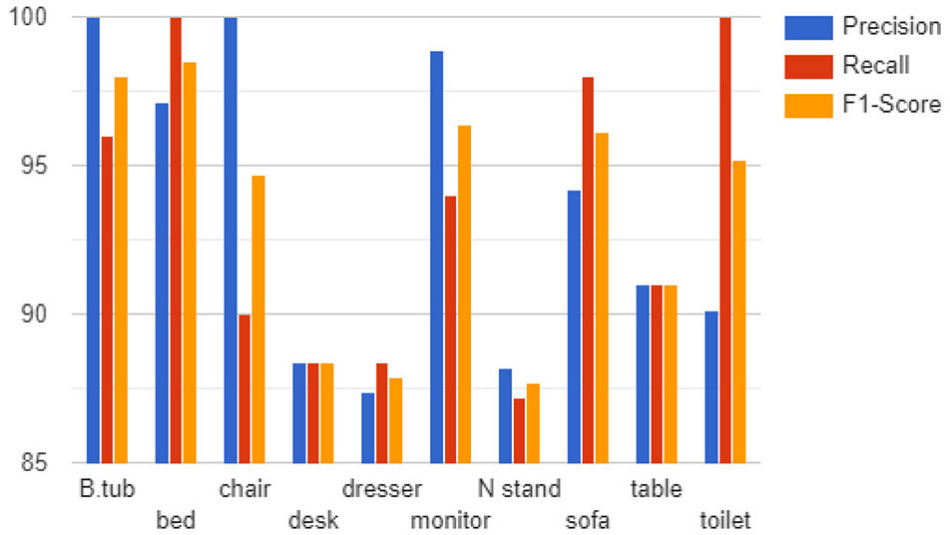
Precision, recall, and F1-score on ModelNet10.

Precision is a metric that assesses the accuracy of predictions, i.e., the percentage of correct predictions. It determines how many of the model's predictions were actually right. The precision was computed using Equation 11 as follows:


(11) $$\begin{array}{l} P=\frac{T_{P}}{T_{P}+F_{P}} \end{array}$$

where *T*_*P*_ is true positive while *F*_*P*_ is false positive (predicted as positive but was incorrect). In the case of recall, it determines how well all of the positives are found which is given as follows:


(12) $$\begin{array}{l} R=\frac{T_{P}}{T_{P}+F_{N}} \end{array}$$

where *F*_*N*_ is false negatives (unable to predict the presence of an object). The mAP is calculated as the average precision of all classes in the dataset while the F1-score is the harmonic mean of the precision and recall. We used these metrics to assess the efficacy and robustness of the proposed method. We used a grid size of 32 × 32 × 32 and kept the value of K at 8. As shown in Figure 6, the model can learn all 10 object class categories with high precision and recall on the ModelNet10 dataset, with 100% precision on bathtub and chair and 100% recall on bed and toilet. We can also observe that the four classes with the lowest precision and recall (desk, table, nightstand, and dresser) are highly similar which makes them difficult to distinguish even by a human expert. As shown in Figure 6, we observed that the proposed approach successfully generated results with (1) more than 90% precision on the bed, monitor, sofa, table, and toilet and more than 80% on the remaining classes, (2) 90% or higher recall of bathtub, chair, monitor, sofa, and table with more than 80% on the desk, dresser, and nightstand, and (3) 90% or higher F1-score of the bathtub, bed, chair, monitor, sofa, toilet, and table with more than 80% on the desk, dresser, and nightstand. This demonstrates that our model can learn discriminative features from 3D shapes directly across several classes.

**Figure 7:**
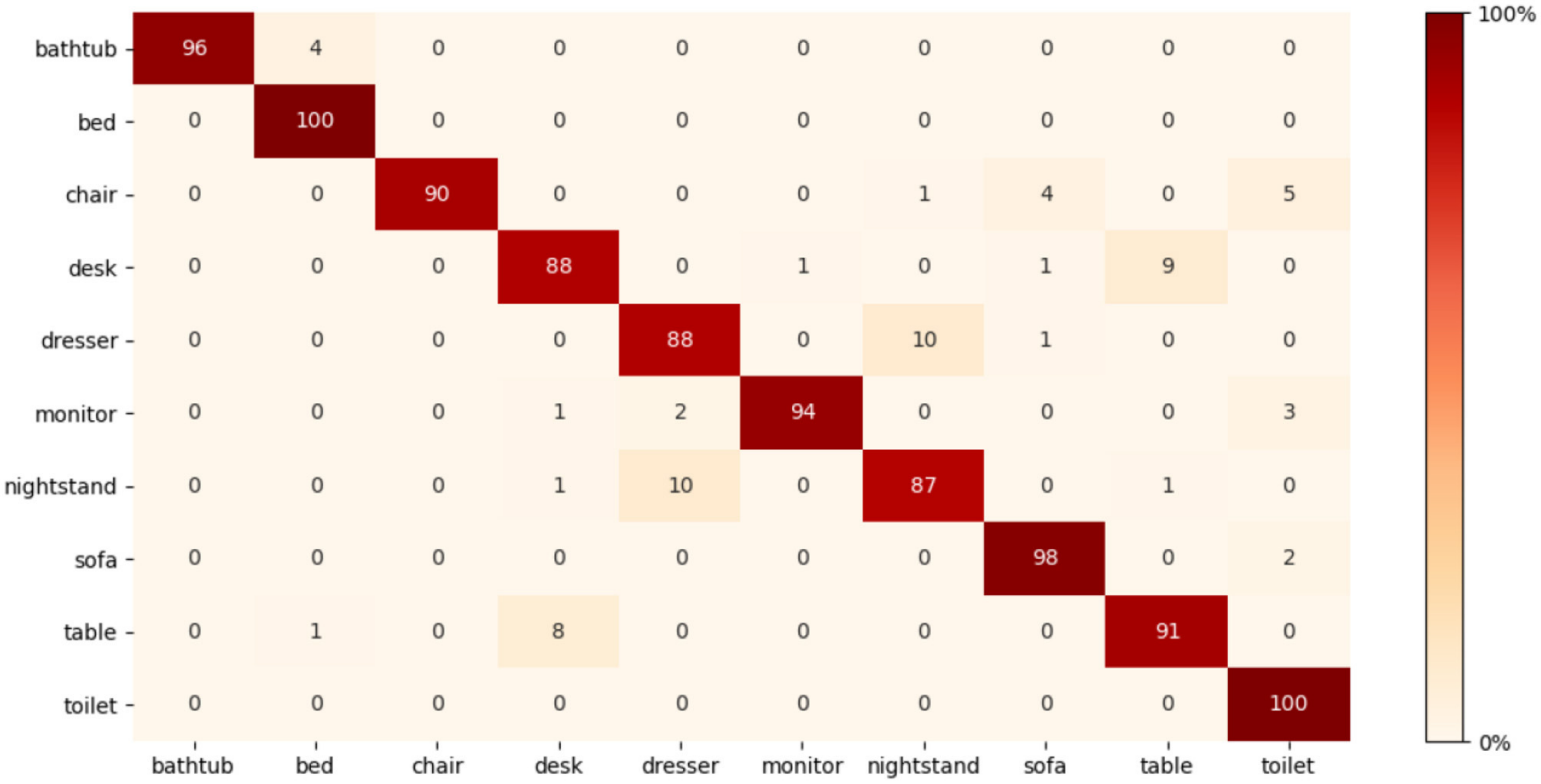
Confusion matrix on ModelNet10.

To calculate the mAP, we perform several experiments, one of which involved using 16 × 16 × 16 voxel size combined with sampling 8 points per grid. The model was trained using ModelNet10 from scratch, which achieved a 90.2% mAP score. We, then, reduced the learning rate by half (0.5^−5^) and retrained the model. The effect of fine-tuning improves the mAP to 90.7%. Another experiment was using a 32 × 32 × 32 grid size with the same points per grid. We train the model using the same procedure in the first experiment. We achieved 92.5% with 0.1^−4^ learning rate, and after reducing the learning rate to half and retraining the model, the result improves to 93.3%. With mAP scores of 93.3%, our model surpasses 3DShapeNets (Wu et al., 2015), PANORAMA-ENN (Sfikas et al., 2017), DeepPano (Shi et al., 2015), PolyNet (Yavartanoo et al., 2021), Multimodal (Chen et al., 2021), SeqViews2SeqLabels (Han et al., 2019), Geometry image (Sinha et al., 2016), and GIFT (Bai et al., 2016) on the ModelNet10 dataset, as shown in Table 4. Even while SeqViews2SeqLabels (Han et al., 2019) has the advantage of pre-existing 2D networks that have been pre-trained on big datasets such as ImageNet1K, we achieved a higher mean average precious mAP with 1.9% margin on ModelNet10. To further illustrate the effectiveness of the improved fused feature network, Figure 7 shows the confusion matrix. The confusion matrix was normalized to 100%. We can see that most objects from all classes are recognized correctly.

**Table 4:** Mean average precision mAP (%) on ModelNet10.

**Method**	**mAP (%)**
3DShapeNet (Wu et al., 2015)	68.3
DeepPano (Shi et al., 2015)	84.1
PANORAMA-ENN (Sfikas et al., 2017)	93.2
SeqViews2SeqLabels (Han et al., 2019)	91.4
Geometry-image (Sinha et al., 2016)	88.4
GIFT (Bai et al., 2016)	91.1
PolyNet (Yavartanoo et al., 2021)	84.6
(Ours) (16 × 16 × 16−*grid*)	**90.7**
(Ours) (32 × 32 × 32−*grid*)	**93.3**

The bold values used to differentiate our results from the rest of the other methods.

### 4.5. Part segmentation on ShapeNetPart

**Figure 8:**
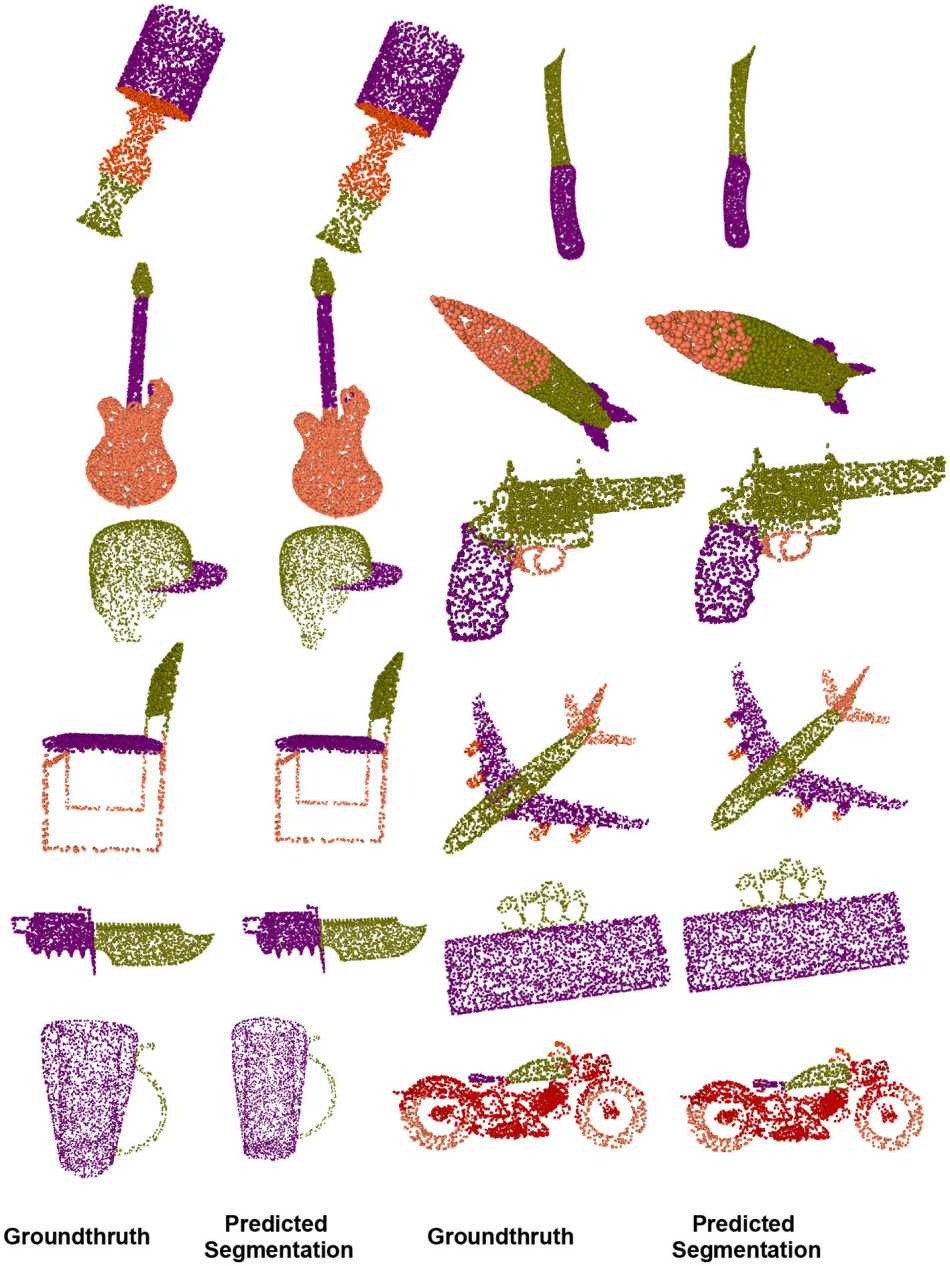
On the ShapeNet-part dataset, we compared the visual results of our object part segmentation with groundthruth.

Part segmentation seems to be more difficult than classification tasks and is regarded as every-point classification. Given a triangular mesh or point cloud representation of a 3D object, the purpose of part segmentation is to give each point or triangle face a part category which makes it more challenging than object classification because of the fine-grained and dense predictions. We used the metric procedure from PointNet++ (Qi et al., 2017). For every part shape within the object category, we calculate the union of prediction and ground truth. Figure 8 shows some ShapeNetPart dataset segmentation results from our method. As observed, in most cases, the proposed method results are visually appealing.

**Comparison:** The segmentation performance of the proposed method is compared with that of various deep learning methods, as shown in Table 5. Although OCNN and RS-Net (Huang et al., 2018) exceed ours in terms of mIoU of all shapes, the improved fused feature residual network outperforms OCNN in specific categories, such as bag, cap, rocket, lamp, and motorbike, and achieves comparable results in the remaining categories. While OCNN has the best IoU, it also uses a conditional dense random field to rectify their network output which serve as a post-processing step, whereas our approach has no similar strategy.

**Table 5:** Segmentation results of different methods on ShapeNet-part dataset (Yi et al., 2016).

**Methods**	**(Ours)**	**P.Net**	**ShapeNet**	**KD-Net**	**MRTNet**	**3DCNN**	**RS-Net**	**O-CNN**
mIoU	**84.2**	83.7	81.4	77.2	83.0	79.4	84.9	85.9
Airplane	83.8	83.4	81	79.9	81.0	75.1	82.7	85.5
Bag	88.9	78.7	78.4	71.2	76.7	72.8	86.4	87.1
Cap	91.9	82.5	77.7	80.9	87.0	73.3	84.1	84.7
Car	72	74.9	75.7	68.8	73.8	70.0	78.2	77.0
Chair	88	89.6	87.6	88.0	89.1	87.2	90.4	91.1
Earphone	47.0	73.0	61.9	72.4	67.6	63.5	69.3	85.1
Guitar	86.8	91.5	92	88.9	90.6	88.4	91.4	91.9
Knife	86.7	85.9	85.4	86.4	85.4	79.6	87.0	87.4
Lamp	89.8	80.8	82.5	79.8	80.6	74.4	83.5	83.3
Laptop	60.8	95.3	95.7	94.9	95.1	93.9	95.4	95.4
Motorbike	93.7	65.2	70.6	55.8	64.4	58.7	66.0	56.9
Mug	94.4	93.0	91.9	86.5	91.8	91.8	92.6	96.2
Pistol	80	81.2	85.9	79.3	79.7	76.4	81.8	81.6
Rocket	86.1	57.9	53.1	50.4	57.0	51.2	56.1	53.5
Skateboard	70.1	72.8	69.8	71.1	69.1	65.3	75.8	74.1
Table	74.1	80.6	75.3	80.2	80.6	77.1	82.2	84.4

The bold values used to differentiate our results from the rest of the other methods.

### 4.6. Ablation experiments

Here, we conduct some ablation experimental tests to assess various setup modifications and highlight the benefits of the improved fused feature network. The experiments were carried out using the ModelNet10 (Wu et al., 2015) dataset.

#### 4.6.1. Effects of extracted features in the DGFE module

**Figure 9:**
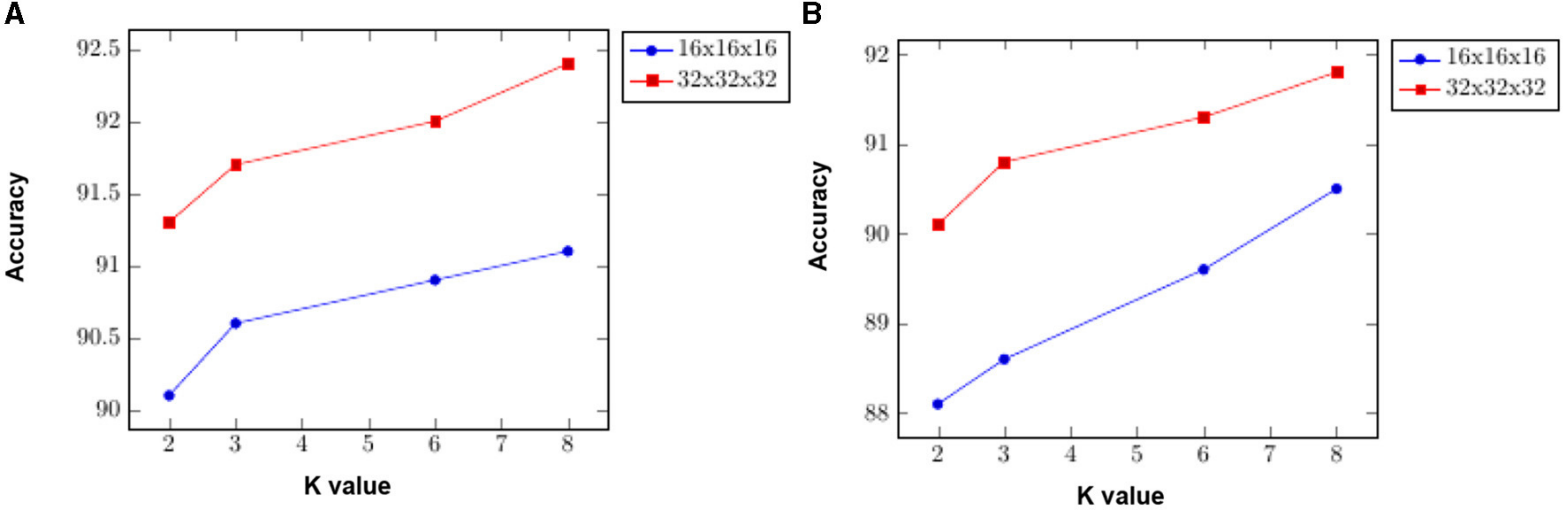
To highlight the influence of both the DGFE module and the feature encoding network, a ModelNet10 classification ablation test is presented. We experimented with some variables including different grid sizes and K values. **(A)** Shows how the feature encoding network performs with 16 × 16 × 16 and 32 × 32 × 32 grid sizes and different values of K; **(B)** demonstrates the performance of the DGFE module's effects of extracted features on 16 × 16 × 16 and 32 × 32 × 32 grid sizes with 2, 3, 6, and 8 K values.

We present an ablation test on ModelNet10 classification to demonstrate the impact of the DGFE module's extracted features. Specifically, we experimented with many variables, including different grid sizes and K values. In the first settings, using a grid size of 16 × 16 × 16 and increasing the value of K from 2 to 8, the classification accuracy increased from 88.1% with K = 2 to 90.5% with K = 8. In the second attempt, we used a grid size of 32 × 32 × 32 and kept the values of K between 2 and 8, and the classification accuracy increased from 90.1% with K = 2 to 91.8% with K = 8. We end up using the later attempt to set the DVFE module in our approach which yields the best model result of 95.6%. Figure 9 displays the results. It shows how the proposed DGFE module encourages correlation among different point cloud regions and is useful for modeling the entire point cloud spatial distribution.

#### 4.6.2. Effects of feature encoding network

This section analyzes the significance of the encoding branch in the proposed approach. After removing the encoding branch, the network is trained using only the DVFE module and KNN search, to sample the local region in each grid cell. We, then, repeated the tests using the same configuration as the previous ablation experiment, with a grid size of 16x16x16 and K = 2. The classification accuracy was 90.1% with K = 2 and 91.1% with K = 8. The classification accuracy improved from 91.3% with K = 2 to 92.4% with K = 8 when utilizing a grid size of 32 × 32 × 32. The results are shown in Figure 9. The model design aids in the efficient encoding of features from the input grid and DVFE module. The output features are combined to complement one another. Figure 9 demonstrates the accuracy achieved by inserting the feature encoding network into the whole network, which results in boosting the classification accuracy. The next experiments investigate the sensitivities of the feature encoding units which consist of two units (Feature Encoding Block FEB Unit A and Feature Encoding Block FEB Unit B) with layer skips containing BN and ReLU in between. In each unit, we start with 3 × 3 × 3 convolutions twice, followed by 1 × 1 × 1 convolutions. The main difference between the units is in the application of BN, a regularly used technique to speed up and stabilize the learning process of deep neural networks, and Relu, which has the advantage of allowing complicated correlations in the data to be learned. To test how resilient our approaches are to changes of this type, we swapped the units in different orders. With a 32 × 32 × 32 grid size and K = 8, we apply four possible combinations, such as ABAB, BABA, AABB, and BBAA. We train the model from the scratch. As shown in Table 6, the classification accuracy is fairly stable across the different combinations. The combination of ABAB has the highest accuracy and the lowest total log loss, with AABB coming in second. Although the two other combinations, BABA and BBAA, have lower accuracy, their overall performance is generally stable. The above result seems to indicate that, in line with He et al. (2016a), adding BN after addition forces skip connections to perturb the output, which is problematic. The main advantage of applying BN before addition here is that it speeds up training and allows a wider range of learning rates without sacrificing training convergence.

**Table 6:** Different combinations of feature encoding units on ModelNet10.

**FEB unit**	**Acc (%)**	**Logloss**
**ABAB**	**95.6**	**2.22**
BABA	93.8	2.38
AABB	94.54	2.25
BBAA	93.94	2.32

#### 4.6.3. Time complexity

Table 7 compares the average testing time for classification and segmentation with other similar methods. TensorFlow 1.1 is used to record forward time using Nvidia Geforce Titan GTX GPU. The proposed method requires less testing time than many other methods, such as (Leng et al., 2016; Charles et al., 2017; Huang et al., 2018), DGCNN (Wang Y. et al., 2019), SpecGCN (Wang et al., 2018), and 3D-UNet (Cicek et al., 2016), because of its strong data closeness and consistency. Because zeros are padded to empty voxel, the proposed voxelization and sampling approaches both include random memory accesses, which help to decrease unnecessary computation. As observed, using the same voxel resolution of 32^3^, the proposed improved fused feature residual network is faster than the 3DCNN (Leng et al., 2016) method and still outperforms it in terms of mIoU, as shown in Table 5. Another advantage of this strategy is that the same number of points is kept in each grid cell while still being able to describe neighborhood information. Now lets analyze the approach to the PointNet++ (Qi et al., 2017), set abstraction module. If we have a batch of 2,048 points with 64-channel characteristics, the technique can model the entire point cloud, but the SA module must aggressively downsample the input, resulting in information loss. The proposed method does not necessitate dynamic kernel computing, which is typically rather expensive. Even though RSNet (Huang et al., 2018) outperformed ours in terms of Mean IoU by 0.7%, the proposed improved fused feature residual network is much faster and requires less memory consumption, as shown in Table 7.

**Table 7:** Average testing time of our method with others on ModelNet40.

**Method**	**Classification (ms)**	**Segmentation (ms)**
PointNet++ (Qi et al., 2017)	163	-
3DCNN (Leng et al., 2016)	49	137
SpecGCN (Wang et al., 2018)	11254	-
DGCNN (Wang Y. et al., 2019)	52	87.8
3D-UNet (Cicek et al., 2016)	-	682.1
RSNet (Huang et al., 2018)	-	74.6
(Ours)	**28**	**19**

The bold values used to differentiate our results from the rest of the other methods.

#### 4.6.4. Effects of neighborhood query

In this section, we experiment with ball query and sift query, two other popular neighbor querying methods to sample local areas and experiment with general search radius. For all experiments, we use a 32 × 32 × 32 grid size with a K = 8 value on the ModelNet10 dataset. Table 8 shows that KNN is more effective for our strategy. The sift query is the most inefficient method when compared with the KNN and ball query.

**Table 8:** Effects of neighborhood query on ModelNet10 classification.

**Sift query**		**Ball query**		**KNN**
***r* = 0.1**	***r* = 0.2**	***r* = 0.1**	***r* = 0.2**	
90.8%	91.0%	92.6%	93.0%	95.6%

## 5. Conclusion and future work

In this study, we proposed the detail grid feature extraction (DGFE) module which is a highly efficient module. This module assists 3D convolutions in hierarchically capturing global information, reducing the grid size in each spatial dimension and managing overfitting by gradually lowering the spatial dimension of the representation, making it practical for high-resolution 3D objects. Furthermore, we design a feature encoding network that uses two different building blocks with layer skips containing batch normalization and non-linearity ReLU in between, resulting in fewer layers in the early training phase which helps speed learning and reduces the effect of gradients vanishing since there are few layers through which to propagate. The outputs of the two modules are fused in the feature fusion unit to produce a feature with improved contextual representation by utilizing both local and global shape structures. We built a network called improved fused feature residual network using the modules that have been proposed, which achieve a notable balance of accuracy and speed. In both ModelNet10 and ModelNet40 datasets, the proposed improved fused feature residual network offers a significant advantage over the bulk of voxel and point cloud-based approaches, as shown in Tables 1, 2. Due to its scalability and efficiency, the proposed method can be used in extracting large-scale features of high-resolution inputs.

Although our method performs well with normal datasets, we note that when noise is added to the datasets, the performance drops, for example, when Gaussian noise is added to the 3D models, the performance decreases despite applying different parameters. In future, instead of directly sampling points, we will use sparse convolutions to convert them to a small number of voxels and sample non-empty voxels to ensure that precise point positions are retained.

In addition, numerous mechanisms for attention employed in transformer approaches are adaptable and offer a high potential for future advances. We think cutting-edge outcomes can be attained by extending generic point cloud processing innovation to transformer techniques. For instance, one possible option we are looking at is by swapping out the feature extraction module in our network design for one that is transformer/attention-based. Instead of just depending on transformers to extract features, we can conduct local feature extraction using non-transformer-based approaches and then couple it with a transformer for global feature interaction which will lead to the extraction of more fine grain features.

## Data availability statement

The raw data supporting the conclusions of this article will be made available by the authors, without undue reservation.

## Author contributions

AG: conceptualization of this study, methodology, writing—original draft preparation, and software. CL: conceptualization, software, supervision, resources, project administration, and funding acquisition. HJ, YN, and MA: data curation, writing—reviewing and editing, and software. HC: data curation, software, and supervision. All authors contributed to the article and approved the submitted version.
